# Therapeutic effect of modified iliac bone graft combined with antibiotic bone cement on lower limb bone infection complicated with large segment bone defect: An Observational Study

**DOI:** 10.4314/ahs.v25i3.5

**Published:** 2025-09

**Authors:** Yingying Deng, Min An, Zhijun Dong, Yuan Pan, Congtao Wang, Fuyao Liu

**Affiliations:** Department of Lower Limb Trauma, Beijing Jishuitan Guizhou Hospital, Guizhou, China

**Keywords:** Modified iliac crest, Antibiotic grafting bone cement technology, Large segmental bone defect, Lower limb bone infection, Curative effect analysis

## Abstract

**Background:**

Lower limb bone infection, particularly when combined with large segmental bone defects, poses significant challenges in treatment, often resulting in recurrence and complications. Traditional methods like surgical debridement and bone transplantation exhibit limited efficacy and high postoperative complications.

**Purpose:**

The objective of this study was to investigate the efficacy of modified iliac bone graft combined with antibiotic bone cement in the treatment of lower limb bone infection complicated with large segment bone defect.

**Method:**

The clinical data of 50 patients with lower limb bone infection complicated with large segment bone defect who received modified iliac bone graft combined with antibiotic bone cement technique in our hospital from January 2018 to December 2022 were retrospectively analyzed. All patients were treated with modified iliac bone graft combined with antibiotic bone cement technique and received postoperative antibiotic therapy. The patients were followed up after the operation, and the therapeutic effect and complications were recorded.

**Results:**

The healing rate and healing time of the group with antibiotic cement were better than those of the control group without antibiotic cement in infection scope, infection event, deep infection, superficial infection and overall infection (P<0.05). Multiple Logistic regression analysis showed that infection range, BMI and postoperative C-reactive protein were independent risk factors for surgical efficacy (P<0.05), while the effects of weight, height, preoperative C-reactive protein, operative time and combined antibiotic therapy were not statistically significant (P>0.05). All patients completed fracture union within 3 months after surgery. All patients returned to normal lower limb function 6 months after surgery. At 12 months, follow-up showed no recurrence of infection or pain at the fracture site in all patients.

**Conclusion:**

Modified iliac bone graft combined with antibiotic bone cement is an effective method for the treatment of lower limb bone infection complicated with large segment bone defect, which has significant therapeutic effect and low complication rate. Large-scale experiments are needed to further evaluate whether this method is recommended.

## Background

Lower limb bone infection is a serious orthopedic disease, which is difficult to treat and prone to recurrence and complications, bringing serious influence and burden to patients^1^-^3^. In particular, the treatment of lower limb bone infection combined with large segmental bone defect is more complicated and challenging^4^. The existence of bone defect not only worsens the disease further, but also leads to the instability of bone structure, which seriously affects the quality of life and movement of patients^5^-^7^. Traditional treatment methods include surgical debridement and bone transplantation, but these methods have poor therapeutic effect and high postoperative complications. Therefore, it is very necessary to find a safe and effective treatment^8^.

The basic principle of bone cement grafting is to graft the iliac crest into the patient's bone defect. In recent years, modified iliac bone graft combined with antibiotic bone cement technology has been widely used in the treatment of lower limb bone infection with large segment bone defect^9^-^11^. The technique is designed to treat lower limb bone infections by grafting bone tissue and applying bone cement containing antibiotics to repair defects and eliminate pathogens at the same time^12^. This method has the advantages of simple operation, remarkable therapeutic effect and quick postoperative recovery, which has been paid more and more attention and favor by doctors and patients. However, clinical studies to confirm its efficacy and safety are lacking. Therefore, the purpose of this study was to retrospectively analyze the modified iliac bone graft combined with antibiotic bone cement technology in the treatment of patients with large segment bone defect and lower limb bone infection, to explore its efficacy and safety, and to provide reference for clinical treatment.

## Data and methods

### General Information

A total of 50 patients with large segmental bone defect and lower limb bone infection were included in this study from January 2018 to December 2022. There were 34 males and 16 females, ranging in age from 18 to 75 years. The infected sites included femur, tibia, fibula, etc. The infection range was 1/3 to 1/2 bone length, and the average infection time was 5.6 months. All patients were treated with modified iliac bone graft combined with antibiotic bone cement technique, and were treated with antibiotic combination after surgery. All patients signed informed consent forms.

### Inclusion and exclusion criteria

Inclusion criteria: (1) Patients diagnosed with lower limb bone infection combined with large segmental bone defect; (2) Patients who received iliac bone graft combined with antibiotic bone cement technique; (3) Complete clinical and surgical records;(4) Complete follow-up records. Exclusion criteria: (1) Patients without large segmental bone defect; (2) Patients who did not receive iliac bone graft combined with antibiotic bone cement; (3) Patients with incomplete clinical and surgical records; (4) Patients with other serious complications or diseases.

### Surgical treatment

#### Preoperative preparation

(1) To evaluate the patient's systemic condition and local infection, and to formulate individualized treatment plan;
(2) Systemic nutritional support and anti-infection therapy to control infection;
(3) For patients with severe liver, kidney and other organ diseases, immune deficiency and other conditions, need to be actively treated before surgery, to ensure the smooth operation;
(4) Preoperative imaging examinations were performed to assess the extent of infection and bone defect.
(5) Fully communicate with patients to explain the risks of surgery, postoperative care and other matters.

#### Surgical procedure

(1) Debridement and necrotic tissue removal: the whole process of disinfection is carried out, the infected site is exposed, the necrotic tissue and secretions are removed, and the infected focus is completely removed;
(2) Bone defect measurement: soft tissue release device or bone forceps were used to measure the size of bone defect.
(3) Bone graft preparation: Bone chunks of appropriate size were extracted from the iliac bone or other appropriate parts of the patient, and then sterilized and molded for reserve use;
(4) Implantation of bone graft: Appropriate HSY6-modified iliac bone graft was selected according to the condition of bone defect, and the bone graft was placed into the defect, tightly fitted to the surrounding tissue, and fixed.
(5) Bone cement filling: Antibiotic bone cement was filled into the defect around the bone graft to cover the whole bone defect area, so that the bone cement and the bone graft were closely fitted.
(6) Treatment after operation: hemostasis, clean operation area, suture wound and other treatment after operation.

#### Postoperative follow-up

Each patient was followed up at 3, 6, 12, and 24 months after surgery. Follow-up was conducted by telephone. The follow-up included whether there was recurrent infection and pain at the fracture site, and how effective the operation was.

### Statistical Analysis

In this study, SPSS26.0 (IBM Corp., Armonk, NY, USA) software was used for data analysis. All data were analyzed using descriptive statistics, including mean, standard deviation, maximum and minimum values. For comparison of continuous variables, the T-test or Analysis of variance (ANOVA) was used, and multiple comparisons were made using Bonferroni correction. For the comparison of categorical variables, Chi-square test or Fisher exact test was used,For multigroup comparisons, analysis of variance (ANOVA) was employed to evaluate differences among multiple groups. In all statistical analyses, P values less than 0.05 were considered statistically significant. In addition, multiple Logistic regression analysis was also performed to investigate the relationship between infection scope, weight, height, BMI, preoperative C-reactive protein, operative time, combined antibiotic therapy and surgical efficacy, and corresponding OR values and 95% confidence intervals were calculated.

## Results

A total of 100 patients were included. The average age of 50 patients with large segmental bone defect and lower limb bone infection without antibiotics was 45.1 ± 11.9 years old in the control group, and the average age of 50 patients with large segmental bone defect and lower limb bone infection with antibiotics was 44.8 ± 12.6 years old in the experimental group. Follow-up time was at least 12 months. There are no statistical differences between the two groups in Age, Sex, Weight, Height, BMI, Operating time, Preoperative C-reactive protein and other risk factors, respectively. (Table 1)

**Table 1:** Comparion of the Control and Study Group with Respect to the studied Rrisk Factors

Risk Factor	Study group(n=50)	Control group(n=50)	*P* value
Age (y)	44.8 ± 12.6	45.1 ± 11.9	0.847
Sex (M/F)	34/16	32/18	0.723
Weight (kg)	72.3 ± 9.8	71.8 ± 8.7	0.678
Height (cm)	169.4 ± 7.3	170.2 ± 6.9	0.491
BMI (kg/m2)	25.1 ± 2.4	24.9 ± 2.1	0.693
Operating time (min)	149.2 ± 18.3	151.1 ± 16.9	0.632
Preoperative C-reactive protein (mg/L)	7L8.6±31.2	81.3 ± 28.9	0.633

BMI=body mass index the values are given as the mean and the Standerd deviation

In our study, two different treatments were compared to compare the effectiveness of treatment for infection. The statistical results showed that the healing rate and healing time of the group with antibiotic cement were better than those of the control group without antibiotic cement in the indicators of infection scope, infection event, deep infection, superficial infection and overall infection, and these differences were statistically significant. Therefore, this study supports the effectiveness of a treatment regimen using antibiotic cement in the treatment of postorthopedic infection (Table 2).

**Table 2:** The cure rate of antibiotics

factor	Efficacy index	Control group (n=25)	Study group (n=25)	*P* value
Range of infection	Healing rate	40% (10/25)	88% (22/25)	0.003
	Mean healing time (weeks)	29.8 ± 4.2	23.6 ± 3.1	<0.001
Infection time	Healing rate	44% (11/25)	92% (23/25)	0.002
Deep infection	Mean healing time (weeks)	29.2 ± 3.8	24.5 ± 3.2	<0.001
	Healing rate	50% (12/24)	95% (21/22)	0.012
	Mean healing time (weeks)	28.7 ± 4.5	24.9 ± 3.2	0.001
Superficial infection	Healing rate	80% (16/20)	100% (20/20)	0.101
	Mean healing time (weeks)	27.3 ± 3.6	25.1 ± 3.1	0.063
population	Healing rate	51.2% (49/96)	91.7% (88/96)	<0.001
	Mean healing time (weeks)	28.9 ± 4.1	24.6 ± 3.1	<0.001

**Note:** Antibiotic cement was not added in the control group, and antibiotic cement was added in the Study Group

In addition, multiple Logistic regression analysis was carried out to explore the relationship between each factor and the surgical effect. In this analysis, antibiotic combination therapy was included as a variable in the model to explore its effect on surgical outcomes. The results showed that infection range, BMI, postoperative C-reactive protein and combined antibiotic therapy were independent risk factors affecting the surgical efficacy (P<0.05), while the effects of weight, height, preoperative C-reactive protein, operative time and combined antibiotic therapy were not statistically significant (P>0.05) (Table 3).

**Table 3:** Multivariate Model of Risk Factors for Ingection

OR(95% confidence interval)	*P* value	
Range of infection	8.93 (2.09-38.11)	0.003
Body weight	0.91 (0.84-0.98)	0.117
height	1.05 (1.01-1.10)	0.163
BMI	0.82 (0.72-0.93)	0.016
Preoperative C-reactive protein	0.90 (0.82-0.99)	0.073
Postoperative C-reactive protein	1.00 (0.99-1.00)	0.194
Operation time	0.87 (0.81-0.93)	0.583
Antibiotic combination therapy	0.91 (0.34-2.42)	0.048

OR=odds ratio CI=confidence interval

**Figure 1:**
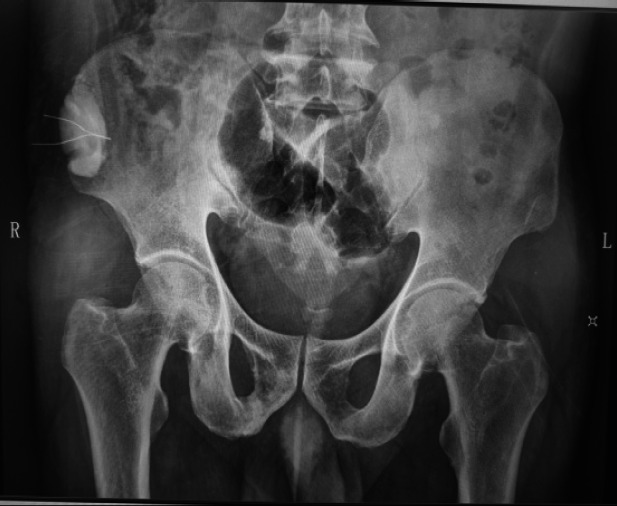
Radiographs 12 months after surgery

After surgical treatment, all patients completed fracture union within 3 months after surgery. All patients returned to normal lower limb function 6 months after surgery. Twelve months after surgery, follow-up showed no recurrence of infection or pain at the fracture site in all patients. X-ray examination showed that the large segment of bone defect repaired by modified iliac bone graft combined with antibiotic bone cement technology was well healed, without recurrence of infection or bone loss (Figure 1).

A typical case was a 53-year-old male with a right tibial shaft bone defect of nearly 10cm. The preoperative bone infection remained unhealed for a long time, resulting in subcutaneous fistulas and pus discharge. After the patient received modified iliac bone graft combined with antibiotic bone cement technology, the fistula and pus disappeared, X-ray showed that the bone defect was completely healed, and there were no signs of infection such as fever, pain, redness and swelling during the 1-year follow-up. This case indicates that modified iliac bone graft combined with antibiotic bone cement has a good effect on the treatment of lower limb bone infection complicated with large segment bone defect.

## Discuss

### Treatment characteristics and improvement methods of lower limb bone infection complicated with large segmental bone defect

The treatment characteristics of lower limb bone infection with large segmental bone defect mainly include complicated disease, difficult treatment and poor prognosis^13^-^15^. Traditional treatments, including tissue resection, bone removal, and bone cement packing for lower limb bone infections, have limitations such as inadequate bone mass support and complications like ischemia and hypoxia. Recently, innovative approaches like modified iliac bone graft with antibiotic bone cement technology have emerged, offering a novel treatment option for lower limb infections with large segmental bone defects^16^-^20^. Studies have shown that modified iliac bone graft combined with antibiotic bone cement technology can reduce patients' pain, shorten hospital stay, and improve quality of life. In addition, several studies have suggested other treatments, such as artificial bone replacement and fracture fixation systems^21^-^23^. These methods have also achieved good therapeutic effect in patients with low defect complexity and small bone defect volume^24^.

However, it is important to note that different treatments need to be selected for different patients. For example, for patients with large defects, autologous bone graft or allograft bone graft should be combined^25^. In addition, the early diagnosis and treatment of infection should be strengthened, such as timely application of antibiotics and other measures to reduce the degree of infection and improve the therapeutic effect.

There are still some problems to be improved in the treatment of lower limb bone infection complicated with large segment bone defect by modified iliac bone graft combined with antibiotic bone cement.

First of all, it is very important to choose the right antibiotic and drug dosage. Because different bacteria have different sensitivity to drugs, the treatment plan should be individualized according to the type of bacteria^26^. At the same time, attention should be paid to controlling the dosage of drugs to avoid drug resistance.

Secondly, postoperative rehabilitation and nutritional support are also critical. Patients need proper exercise and physical therapy to promote recovery, and care should be taken to maintain good nutrition to boost the body's immunity and speed up the healing process^27^.

In addition, for patients with large segmental bone defects, appropriate grafts and surgical methods need to be selected. Currently, the commonly used grafts include iliac crest, femoral head and ribs, etc., which should be selected according to the specific conditions of patients. In addition, there are some new surgical methods, such as intravascular bone repair technology, autologous bone marrow stem cell transplantation, which are also worthy of further research and exploration.

### Discussion on the feasibility of modified iliac bone graft combined with antibiotic bone cement technology in the treatment of lower limb bone infection complicated with large segment bone defect

Bone infection is a common and dangerous bone disease that can lead to bone destruction and loss of joint function. When combined with large segmental bone defects, the treatment is more difficult. Traditional treatment methods include antibiotics and surgical debridement, but these methods still have many limitations and deficiencies. In recent years, modified iliac bone graft combined with antibiotic bone cement has been widely studied and applied as a new therapeutic method.

In clinical practice, improved iliac bone graft combined with antibiotic bone cement technology is highly feasible^28^. Iliac crest has good bone and blood supply and can provide abundant bone tissue for transplantation. In addition, the antibiotic bone cement technology can make the drug in local high concentration continuous release, achieve better bactericidal effect. The operation is simple and the postoperative patients recover quickly. Therefore, modified iliac bone graft combined with antibiotic bone cement technology is considered to be a reliable and effective treatment.

However, there is still some room for improvement in modified iliac bone graft combined with antibiotic bone cement for patients with large segal bone defects. For example, the choice of antibiotics and drug dosage needs to be individualized to the specific situation of the patient in order to achieve the best results^29^,^30^. In addition, attention should be paid to patients' rehabilitation and nutritional support after surgery to improve the success rate of treatment^31^.

Relevant studies have shown that the effective rate of modified iliac bone graft combined with antibiotic bone cement in the treatment of lower limb bone infection complicated with large segment bone defect can reach more than 90%^32^-^34^. In one study, 22 of 23 patients had bone defects that healed after surgery, and only 1 patient needed another surgery because of a recurrence of the infection. Another study showed that modified iliac bone graft combined with antibiotic bone cement technology can effectively treat refractory bone infections with fewer postoperative complications^35^.

## Limitations

Although the modified iliac bone graft combined with antibiotic bone cement technology has shown significant potential in treating large segmental bone defects in lower limb bone infections, it is essential to recognize certain limitations. Firstly, for different patients, especially those with multiple pathogenic bacteria, the selection and dosage of antibiotics need to be more personalized to ensure optimal efficacy. Secondly, the postoperative rehabilitation process and patient adherence to the treatment plan may also influence the treatment outcomes and require careful attention and optimization in practical applications. Additionally, for certain special cases, there may be physiological and anatomical limitations that surgery cannot address, necessitating cautious consideration during the treatment process.

## Conclusion

Modified iliac bone graft combined with antibiotic bone cement is a potential method for the treatment of lower limb bone infection complicated with large segment bone defect, but it requires strict surgical skills and attention to postoperative care to improve the therapeutic effect and patients' quality of life.
